# The client satisfaction with device: a Rasch validation of the Arabic version in patients with upper and lower limb amputation

**DOI:** 10.1186/s12955-021-01773-1

**Published:** 2021-04-27

**Authors:** Hadeel R. Bakhsh, Nilüfer Kablan, Walaa Alammar, Yaşar Tatar, Giorgio Ferriero

**Affiliations:** 1 Department of Rehabilitation, College of Health and Rehabilitation Sciences, Princess Nourah Bint Abdulrahman University, Riyadh, Saudi Arabia; 2 Department of Physiotherapy and Rehabilitation, Faculty of Health Sciences, Istanbul Medeniyet University, Istanbul, Turkey; 3 Faculty of Sports Sciences, Marmara Üniversity, Istanbul, Turkey; 4 Physical and Rehabilitation Medicine Unit, Scientific Institute of Tradate, Istituti Clinici Scientifici Maugeri IRCCS, Tradate, Varese Italy; 5 Department of Biotechnology and Life Sciences, University of Insubria, Varese, Italy

**Keywords:** Rasch analysis, Prosthesis, Patient satisfaction, Psychometrics, Rehabilitation

## Abstract

**Background:**

The Client Satisfaction with Devices (CSD) module of the Orthotics and Prosthetics Users’ Survey is an extensively used questionnaire that measures patients’ satisfaction with orthosis and prosthesis. However, the validated version for Arabic speakers (CSD-Ar) is only applicable for orthosis users.

**Objectives:**

The aim of this study was to evaluate the psychometric proprieties of the CSD-Ar for prosthetics users.

**Methods:**

The study used a convenience sample of prosthesis users from Saudi Arabia and Turkey (N = 183), who completed the CSD-Ar. The collected data were analysed using Rasch analysis to evaluate item fit, reliability indices, item difficulty, local item dependency, and differential item functioning (DIF) using WINSTEPS version 4.6.1.

**Results:**

Based on the analysis, the four-response Likert-scale was acceptable, as shown by the category functioning test, All eight items did achieve a fit to the Rasch Model [(infit) and (outfit) mean-square 0.75 to 1.3]. Person separation reliability was 0.76, and item separation reliability was 0.94. A principal component analysis (PCA) showed satisfactory unidimensionality and no local item dependency. The DIF analysis showed no notable dependency among items on participant characteristics in terms of age, gender, duration of use, country, and level of amputation.

**Conclusion:**

This study contributes to the confidence of using CSD-Ar to evaluate users’ satisfaction with different prostheses, affirming the need for further refinement of the quality of the outcome measure.

## Introduction

Limb amputation is a major cause of long-term disability. The incidence of amputation varies in different countries, ranging between 1.2 and 4.4 cases per 10,000 inhabitants per year. It is estimated that the demographic changes and increasing incidence of diabetes might double the number of amputations over the next 30 years [1].

Assistive devices play a significant role in rehabilitation and ensuring an enhanced quality of life for individuals who undergo amputation [2]. In patients who have undergone limb amputation, assessment of patient satisfaction with psychometrically comprehensive measures is an important factor along with other evaluations in clinical decision making [3].

Few tools have been validated for the assessment of patient satisfaction with assistive devices among individuals living with an amputated limb [4]. Among these, Client Satisfaction with Device (CSD) is one of the most widely used tools [5]. The CSD is one of the five modules of the Orthotics Prosthetics Users Survey. It is a self-report outcome measurement tool for people using prostheses and orthoses [5]. The original CSD version comprised 11 items and has undergone significant revision using the Rasch analysis (RA). Three items were subsequently excluded as they were inappropriate and/or belonged to a different constructs [5–7]. The adjusted CSD questionnaire has eight items and uses a four-point Likert scale, which is widely considered the most accurate version to date [7–9].

As the demand for both healthcare service quality and patient satisfaction increases, there is a need for psychometrically reliable measures owing to their increasing role in decisions that enhance the prescription, policymaking, and expenditure of prosthetics. RA provides a comprehensive psychometric investigation of the tool in question as it is a widely accepted rigorous method in the assessment of rating scales, offering psychometric information that cannot be acquired using the classical test theory [10].

The CSD questionnaire was initially developed in English and validated among users of prosthesis across several cultural backgrounds, mainly individuals residing in Western countries. In these countries, peripheral vascular diseases account for 80–90% of all amputations, and the rate of amputations due to traumatic accidents has been constant or declining [6, 9, 11, 12]. However, in other countries, such as Arabic-speaking countries, trauma is the main cause of amputation, particularly from road traffic accidents, and patients tend to be young adults [13–17]. Furthermore, in some of these countries, owing to wars and the existence of landmines, an increasing rate of amputations has been observed [12, 18]. In Arabic-speaking countries, there are no validated outcome measures for user satisfaction in terms of their experience with the use of prosthesis. The Arabic version of the CSD questionnaire (CSD-Ar) is available but has been validated among orthosis users [8]. To date, no study has been conducted to investigate the psychometric characteristics of the CSD-Ar questionnaire among Arabic-speaking prosthesis users [6].

In response to the need for a validated and psychometrically reliable outcome measures for prosthesis users in these countries, this study aimed to validate the psychometric properties of the CSD-Ar questionnaire through advanced modern psychometric analyses such as RA among Arabic-speaking prosthesis users with upper- and lower-limb amputations.

## Methods

### Patients

**Fig. 1:**
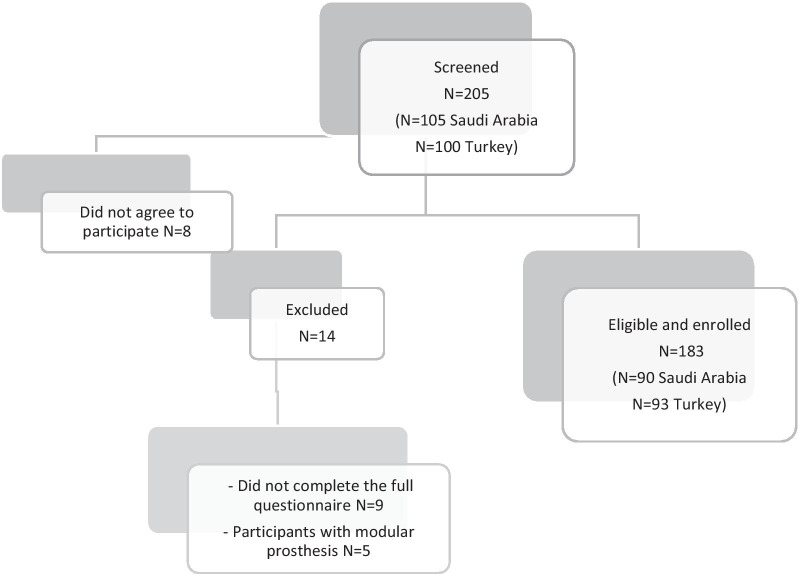
Enrolment process for CSD-Ar: flowchart

Patients were progressively recruited from two countries, Saudi Arabia and Turkey, between August 2017 and August 2019. Two hundred and five patients were asked to participate, of which 183 agreed (Fig. 1). In Saudi Arabia, 90 patients were recruited from rehabilitation department referrals from three medical institutions in Riyadh. In Turkey, 93 patients were recruited from three prosthesis and orthosis centres in Istanbul, Reyhanli, and Sanliurfa, respectively. These centres are funded by the Zakat House of Kuwait and established and managed by the Alliance of International Doctors, an international non-governmental organisation that provides prosthetic and orthotic services to Syrian refugees in Turkey who underwent amputations because of injuries in the Syrian civil war or owing to accidents or medical complications.

### Inclusion and exclusion criteria

The inclusion criteria included current use of a prosthesis for at least 6 months, age ≥ 18 years, and a native Arabic speaker. The exclusion criteria included the inability to communicate (read) in Arabic, prosthesis use for less than 6 months, and diagnosis of a cognitive deficit (Mini-Mental State Examination score < 28).

### Client satisfaction assessed using the CSD questionnaire

The CSD questionnaire pertains to several orthosis/prosthesis-related parameters (e.g., weight, aesthetics, and comfort). The adjusted CSD edition comprises eight items assessed using a four-point Likert scale: 1 (‘strongly disagree’) to 4 (‘strongly agree’). Higher scores indicate higher agreement/satisfaction [5, 7]. The CSD questionnaire was translated and adapted cross-culturally for Arabic-speaking individuals (CSD-Ar questionnaire) and validated in this population only for orthosis users [8].

### Statistical analysis

Measure of central tendencies are used to outline the cohort’s demographics and clinical characteristics. For continues variables the mean and standard deviation was used, and for categorical variables the median was used. The use of modern psychometric models such as the RA are increasingly being used in the validation of clinical outcome measures as they are a meticulous statistical method, far superior to classical test theory because they are able to determine to what extent the measure adheres to psychometric requirements to produce a sound measurement [10, 19]. WINSTEPS® Software (Version 4.6.1, Winsteps.com, Beaverton, OR, USA) was used to conduct the RA to examine the following [20]:

(a) *Rating scale diagnostics* The CSD-Ar was evaluated using the guidelines recommended by Linacre [21] as follows: (1) a minimum of 10 observation for each category; (2) even distribution of category frequencies (3) category outfit mean square values of < 2; (4) monotonic increase in both average measures of persons with a given score/category and thresholds (threshold is the ability at which response to either of two adjacent categories is likely; and (5) threshold difference > 0.81 log-odd and < 5.

(b) *Construct validity* The evaluation of items fit to the latent trait conducted by inspecting the pattern of item difficulties if consistent with expectations of the mode [22]. Information-weighted (infit) and outlier-sensitive (outfit) mean-square statistics (MnSq) for each item were computed, and an acceptable fit was defined based on the sample size as MnSq ranges from 0.75 to 1.3 [22]. The presence of larger values for items is considered misfitting (signalling unexpectedly high variability), whereas smaller values indicates overfitting (signalling predictable pattern) [10]. Additionally, the estimates of item difficulty and the location of single subjects across an interval scale were calculated. The item difficulty and subject ability are indicated in logit units. A cohort size of a minimum of 150 participants allows for a stable calibration of items within ± 0.5 logit and 95% confidence [23].

(c) *Reliability* This measure is evaluated in terms of (G), which is defined as the ratio of the true spread of the measures to their measurement error [22]. A person-separation index of 2.0 reflects an ability to distinguish between three levels, or ‘strata’, of measures statistically marked and comparable to a reliability of 0.80 (interpreted similarly to Cronbach's alpha) [24].

(d) *Dimensionality and local dependency* A principal component analysis (PCA) of standardized residuals was performed in order to evaluate: (1) the presence of sub-dimensions, as an independent confirmation of the unidimensionality of the scale, assuming that the residuals will be uncorrelated and normally distributed; to conclude if other factors were likely to be present in the residuals, the following principles were adopted: (a) a cut-off of 50% of the variance explained by the Rasch factor and (b) eigenvalue of the first contrast < 2; and (2) the local independence between items, considering that item couples with a standardized residual correlation higher than (0.30) as possibly dependent components [20].

(e) Differential Item Functioning (DIF) investigated if the items had similar difficulty hierarchies across all demographic groups and explored probable differences due to context effects between measures obtained across split subgroups. This research outlined five dichotomous categories based on (1) gender (male vs. female), (2) affected region (lower vs. upper limb), (3) duration of use (6 month-to-a year vs. more than one year, (4) country (Saudi Arabia vs. Turkey), and (5) age (younger vs. older, spliting the cohort at median age of 36 years old). Pairwise item-by-item difficulty DIF tests between the two sets were computed (two-sided t-test) for the differences between means. A prior assumption was that the authors would not find DIF between the analysed groups. To detect DIF, a minimum of 0.5 logit difference with a p-value < 0.05 was used as criterion [25].

### Procedure and ethical approvals

Approval was obtained from the local Institutional Review Board for Ethics as well as from participating hospitals in Saudi Arabia and Turkey. This study fulfilled the regulations and requirements delineated in the Declaration of Helsinki. The patients were recruited prior to or after a follow-up visit by clinicians (physical and occupational therapists). The clinicians explained the questionnaire and purpose of the study, and the respondents agreed to participate by signing a consent form. The clinicians involved in collecting the data were not responsible for the patients’ care nor were they affiliated with the selected rehabilitation institutions of the study.

## Results

Questionnaires submitted were complete and had no missing responses from patients. The demographics, clinical characteristics, and measurement scores of the cohort (n = 183) are shown in Tables 1 and 2. Eleven (6%) patients had a minimum score (floor response), and one patient (0.5%) had the maximum score (ceiling response).

**Table 1:** Participants’ demographic and clinical characteristics

	Saudi Arabia N = 90		Syrian Refugees in Turkey N = 93		Total N = 183	
Age (years mean (SD))	34.6 (± 14)		38.4 (± 15)		36.51 (± 14)	
Gender	n	%	n	%	n	%
Male	77	86	86	92	163	89
Female	13	14	7	8	20	11
Education	n	%	n		n	%
Uneducated	5	6	5	5	10	5
Elementary	4	4	27	29	31	17
Secondary	9	10	28	30	37	20
High school	24	27	22	24	46	25
Undergraduate	43	47	1	1	44	24
Graduate	5	6	10	11	15	8
Employment	n	%	n	%	n	%
Unemployed	24	27	19	20	43	23
Student	10	11	10	11	20	11
Military	6	7	0	0	6	3
Private sector	22	24	29	31	51	28
Governmental sector	19	21	10	11	29	16
Other	9	10	25	27	34	19
Cause of amputation	n	%	n	%	n	%
Traumatic	56	62	76	82	132	72
Non-traumatic	34	38	17	18	51	28
Duration of use	n	%	n	%	n	%
6 months-a year	25	28	63	68	88	48
1–5 years	32	36	30	32	62	34
5–10 years	14	16	0	0	14	8
Over 10 years	19	21	0	0	19	10
Affected region	n	%	n	%	n	%
Right lower limb	40	44	35	38	75	41
Left lower limb	32	36	41	44	73	40
Bilateral lower limb	12	13	4	4	16	9
Right upper limb	0	0	0	0	0	0
Left upper limb	1	1	7	8	8	4
Bilateral upper limb	5	6	6	6	11	6
Level of amputation	n	%	n	%	n	%
Transhumeral	5	6	4	4	9	5
Elbow disarticulation	1	1	0	0	1	1
Transradial	0	0	10	11	10	5
Transfemoral	41	46	35	38	76	42
Knee disarticulation	1	1	1	1	2	1
Transtibial	41	46	39	42	80	44
Ankle disarticulation	1	1	0	0	1	1
Chopart	0	0	4	4	4	2
Prosthesis type	n	%	n	%	n	%
Foot prosthesis	2	2	0	0	2	1
Standard below knee	38	42	42	45	80	44
Microprocessor knee prosthesis	25	28	2	2	27	15
Upper Limb Cosmetic prosthesis	2	2	0	0	2	1
Polycentric hydraulic knee	16	18	6	6	22	12
Rushfoot	1	1	0	0	1	1
Myoelectric Prosthesis	3	3	0	0	3	2
hybrid upper extremity prosthesis	1	1	0	0	1	1
Smart Ankle	**1**	1	0	0	1	1
Pneumatic knee	**0**	0	16	17	16	9
Harness system prosthesis	**0**	0	14	15	14	8
Monocentric knee	**0**	0	13	14	13	7

**Table 2:** Raw measurement scores of CSD-Ar

Items	Saudi Arabia N = 90 Mean (SD)	Syrian refugees in Turkey N = 93 Mean (SD)	Total N = 183 Mean (SD)
1. My device fits well	1.6 (.66)	1.8 (.78)	1.7 (.73)
2. The weight of my device is manageable	1.9 (.81)	1.9 (.70)	1.9 (.76)
3. My device is comfortable throughout the day	2.2(.84)	2.3 (.96)	2.3 (.90)
4. It is easy to put on my device	1.7 (.74)	1.9 (.81)	1.8 (.78)
5. My device looks good	1.9 (.82)	2.1 (.86)	2.0 (.84)
6. My device is durable	1.6 (.66)	1.9 (.79)	1.8 (.74)
7. My skin is free of abrasions and irritation	2.1 (.93)	2.2 (.92)	2.2 (.92)
8. My device is pain free to wear	2.0 (.90)	2.2 (.92)	2.1 (.91)
Total	15.2 (4.07)	16.4 (4.42)	15.8 (4.28)

Rating-scale diagnostics using RA demonstrated that the four-point scale complied with the benchmarks stipulated in terms of the monotonic rise in average category measures and relevant thresholds, as shown in Table 3. Moreover, the respective difference in threshold was between one and five logits, with an outfit mean-square value less than two.

**Table 3:** Category functioning for CSD-Ar

Category label	Category measure	Andrich threshold	Outfit* MnSq	Infit** MnSq***	Category response frequency (%)
1 Strongly disagree	− 3.19	NONE	1.05	1.13	446 (30%)
2 Disagree	− .76	− 2.05	.91	.85	702 (48%)
3 Agree	1.07	.76	.96	.92	221 (15%)
4 Strongly agree	2.68	1.29	.99	1.01	95 (6%)

Average category measures, thresholds, category fit statistics, and observed frequency (count) for the four-category rating scales of the OPUS CSD-Ar Module. A monotonic increase in both average measures across rating scale categories was observed, thresholds increased, category outfit mean square values were less than 2, and the number of observations per category was appropriate

^*^outlier-sensitive

^**^information-weighted

^***^mean square

**Fig. 2:**
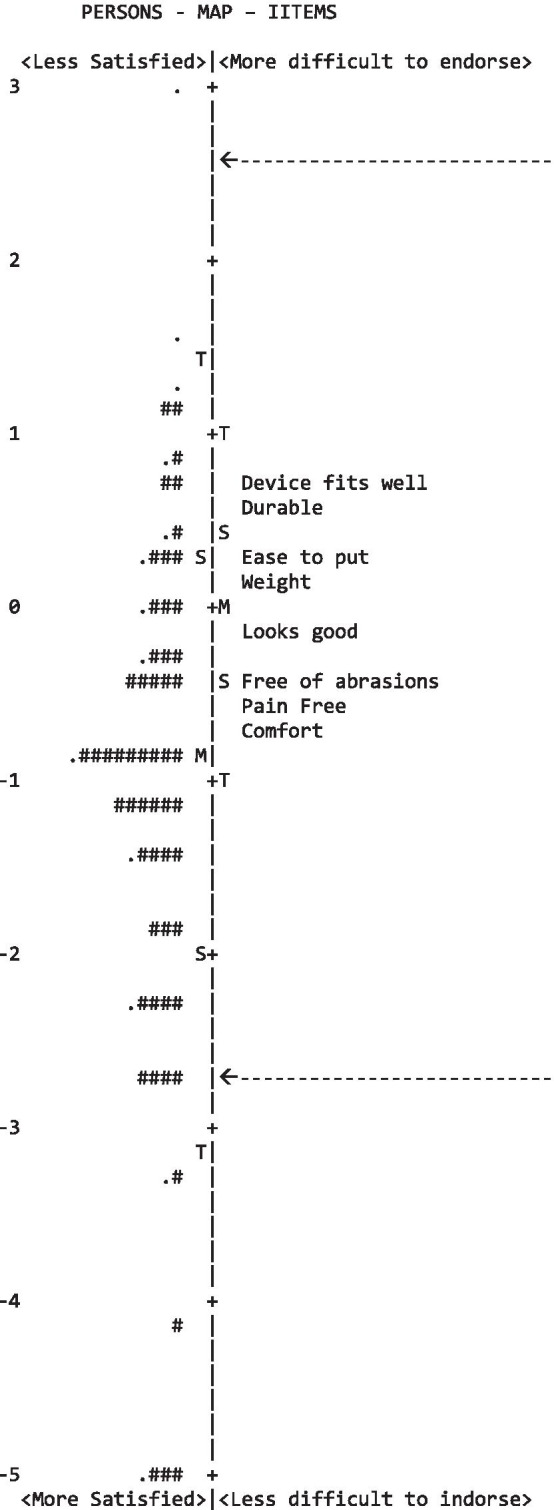
Wright map: the subject-ability and item-difficulty map of the Arabic CSD module. The line represents the measure of the construct *satisfaction with device* in linear logit units with average difficulty of items set to 0 (indicated by M). On the left column is the distribution of individual’s “ability/agreement” along the construct (satisfaction): each "#" denotes three individuals while "." denotes one to two individuals. Top to bottom measures indicate lower to higher satisfaction with device. On the right column is the item difficulty measure for each category along the construct based on the rating scale model. The higher the item estimate, the more difficult the item was for the cohort to endorse/agree with (i.e., showing lower scores and indicating less satisfaction with this item). The highest and lowest item response category step calibrations are indicated with arrows

In terms of item misfit statistics, Table 4 illustrates that all eight CSD-Ar questionnaire items fit the fundamental construct that the scale intends to measure. The CSD-Ar questionnaire showed a mistargeting item difficulty, which is presented in the Wright map of patient satisfaction and item difficulty for the eight items on the same logit scale (Fig. 2).

**Table 4:** Item calibration (measure increasing bottom up)

Items	Measure (SE)	Infit	Outfit
		MsSq	MnSq
1. My device fits well	.68 (.13)	.88	.85
6. My device is durable	.55 (.13)	1.08	1.09
4. It is easy to put on my device	.34 (.12)	1.03	.99
2. The weight of my device is manageable	.21 (.12)	.96	1.03
5. My device looks good	− .16 (.12)	1.07	1.04
7. My device is pain free to wear	− .42 (.11)	.99	.94
8. My skin is free of abrasion and irritation	− .51 (.11)	1.22	1.21
3. My device is comfortable throughout the day	− 0.70 (.11)	.76	.75

Patients’ ability levels covered 10.09 logits (from − 5.40 to 4.69; average measure − 1.14), and item difficulty estimates covered 1.38 logits (from − 0.70 to 0.68). Table 4 demonstrates that a higher item measure (i.e., Item #1 ‘*My device fits well’*) shows lower scores (using a rating scale from 4 ‘strongly agree’ to 1 ‘strongly disagree’), representing low satisfaction with the prosthetic feature of “fit”. Meanwhile, item #3, namely ‘*My device is comfortable throughout the day*’, was the easiest item to endorse (higher scores).

With the measure increasing bottom up (from negative to positive values), the more difficult item was for the participants to endorse (i.e., showing lower scores and indicating less satisfaction with this item). Each item estimate can be regarded as the balancing point for the response distribution across that item’s categories. Acceptable fit is defined as MnSq 0.75 to 1.3.

In terms of reliability, the indices were as follows: person-separation index = 1.78 with person-separation reliability = 0.76 (Cronbach’s α = 0.80); Item-separation index = 3.84 with item-separation reliability = 0.94. These values allow researchers to distinguish two ability strata (satisfied vs unsatisfied).

The outcomes of the PCA analysis showed satisfactory unidimensionality, with a variance explained by Rasch measure of 38.7% (eigenvalue 5.50). Moreover, the eigenvalue of the first contrast was at 1.91 (unexplained variance of 14.7%). The correlation between item residuals was lower than 0.30, indicating the absence of local dependency on items.

Finally, the DIF analysis showed no notable dependency of the measure on cohort characteristics, with a smaller DIF contrast. In the DIF analysis, the sample size range was as follows: sex (− 0.27 to 0.56), affected limb (− 0.46 to 0.70), age of the patient (− 0.17 to 0.18), country of the patient (− 0.20 to 0.21), duration of use of prosthetics (− 0.16 to 0.16) (DIF size would have to be > 0.8 logits to be significant at 0.05% confidence level in our cohort).

## Discussion

This study verified the validity of the structure of the Arabic version of the eight-item CSD questionnaire for prosthesis users, based on the fit indices and measurement of non-invariance shown by the absence of DIF [7]. This was the first study in which the CSD-Ar questionnaire was validated among Arabic-speaking populations that included both types of subjects—users of the lower-limb and users of an upper-limb prostheses—to evaluate their satisfaction. Previous studies involving CSD questionnaires have been conducted on cohorts comprising only upper-limb or only lower-limb prosthesis users [6, 11], while those using CSD-Ar questionnaires involved users from a single Arabic-speaking country and of othrosis only [8].

This test confirmed the adequate functionality of the four-point scale categories when tested using RA, with a well-perceived difference between the four points by the patients. Moreover, the fit statistics indicated that all eight items fit the model. The results of this study are comparable with those of Burger et al., who evaluated the Slovenian CSD questionnaire on upper-limb prosthesis users with infit and outfit MnSq values 0.60–1.40, based on a sample size of 76 [11].

In this study, the targeting of item difficulty to the participants’ ability/agreeablity (the array in which the items are appropriately difficult for the cohort) was poor, as illustrated in Fig. 2. When comparing with the mean value of 0 logits that is normally assigned for items, the cohort’s average satisfaction levels (− 1.14) logit was higher than the difficulty levels of these items. These findings contradict the results of Bakhsh et al. [8] in which the CSD-Ar questionnaire was initially validated among orthotics users; their results showed a reasonably well-targeted patient-ability with a mean measure of − 0.89. However, this study’s results are in line with those of Burger et al. [11] and Bravini et al. [9] who found comparable values of − 1.39 and − 1.64, respectively, for item difficulty when administered to prosthetic and orthotic users. These findings can be anticipated from a clinical perspective. Patients' experiences with orthoses might differ from those using prostheses and their reason for use might be different [26, 27]. Furthermore, from a psychological perspective, trauma might affect how the participants feel post-amputation, leading them to express gratitude towards the care provided [27]. However, this mistargeting of item difficulty indicates that benefits can be derived from higher levels of satisfaction during prosthetic device analysis. Therefore, future studies should test whether the addition of more difficult items would enhance the questionnaires’ construct and quality.

Furthermore, the item-difficulty hierarchy is different from that published in existing studies because previous psychometric studies of CSD questionnaires included patients from different cultures, wearers/users of different devices, and variations in the availability of devices. Therefore, considering these variables, participsants perceived items in the questionnaire differently in terms of their importance and difficulty. For example, for participants in this study, items related to “fit” had a lower score of satisfaction than those in the study by Bakhsh et al. and Bravini et al. [8, 9]. These contradictory findings stem from factors influencing satisfaction with the fit (material, duration of use, and functionality, among others). Therefore, a comprehensive comparison of the results is not feasible since these studies included different sample populations and devices (i.e., orthoses and prostheses).

In terms of reliability, the CSD-Ar questionnaire results are in line with those of previous studies that used the classical test theory methodology in addition to RA, as noted by Hadadi et al. and Burger et al., who reported Cronbach’s alpha values of 0.71 and 0.76, respectively [7–9, 11, 28]. These results might be indicative of the effect of the value of the tool in the context of group decisions pertaining to patient satisfaction rather than clinical usage in a single-user context, for which it is preferable to attain a minimum 0.90 reliability threshold [29].

The unidimensionality of the CSD-Ar questionnaire based on the PCA of standardised residuals is in line with previously published results [8, 9, 11]. Though the values were relatively high, they did not present significant residual load on extraneous factors (unexplained variance < 15% with eigenvalues of the first contrast < 2). [20, 22, 30–32] These results might indicate essential unidimensionality however, this is not a definite unidimensionality. One of the main reasons for the weak unidimensionality is a possible contrast between the items related to comfort (the main factor) of the devices and those related to appearance and durability [6].

Though a multicenter approach from two countries, including all types of prosthesis, was used in this study, caution is necessary when generalising the findings to other groups. The ability to generalise specifically relates to the criteria for selection of the convenience sample. The duration of prosthesis use was less than 1 year in almost half of the sample because the study recruited many Syrian refugees, who had only recently received their first prosthesis. This group of participants showed satisfaction levels similar to those of the Saudi group, who had a longer duration of prosthesis use. This finding is in line with those in previous studies, showing that time since limb loss did not significantly correlate with satisfaction with the device, even among young amputees [33].

Our study had some limitations. The sample population had an uneven distribution in terms of both sex and region of amputation (the sample comprised male lower-limb amputees predominantly). However, this distribution of the sample demographics is common in this part of the world, with similar sample distribution reported by Ali et al. and Destile et al. [34, 35].

Another limitation was the lack of consideration of potential DIF in devices among the different types of prostheses. This limitation might be particularly important considering that Saudi patients had access to a variety of prostheses (i.e., myoelectric) different from that for Syrian patients, who received limited types of prostheses. Furthermore, under Turkish healthcare, materials and production of the prostheses were the same; Prosthesis fitting and examination were performed by a single specialist before being delivered to the patient. Through this method, standard quality and homogeneity were maintained in the production and delivery of prostheses for patients in Turkey, but not in Saudi Arabia. Notably, in Saudi Arabia, the national health system reimburses the expenses for prostheses for all ages (in most cases) and provides the choice of various prosthetic designs (i.e., myoelectric) at no additional cost to the patient.

## Conclusion

This study provides further evidence of the psychometric properties of the CSD-Ar questionnaire and its suitability for use in various clinical settings to includ prosthesis users. The availability of this outcome tool in Arabic-speaking clinical settings would be beneficial in enhancing the quality of rehabilitation services provided for prosthesis users. Moreover, this study provides suggestions for further refinements and supplementary tests to the CSD-Ar questionnaires to extend its validity across different cultural backgrounds, age groups, and prosthesis types.

## Data Availability

The datasets during and/or analysed during the current study available from the corresponding author on reasonable request.

## Abbreviations

OPUS: Orthotics and Prosthetics User’s Survery
CSD: Client satisfaction with device
RA: Rasch analysis
INFIT: Information-weighted
OUTFIT: Outlier-sensitive
MNSQ: Mean-square statistics
DIF: Differential item functioning
